# Supplementary material for: Deletion of FNDC5/irisin modifies murine osteocyte function in a sex-specific manner
Source: eLife. 2024 Apr 25;12:RP92263. doi: 10.7554/eLife.92263 (PMC11045224; doi:10.7554/eLife.92263)
Supplement: Supplementary file 1. — Femoral cortical and trabecular bone parameters of wildtype (WT) and FNDC5 KO female virgin and lactation mice. n = 5–8/group. Data presented as mean ± standard deviation. a=Significant compared to WT control, b=significant compared to KO control, c=significant compared to WT low-calcium diet, two-way analysis of variance (ANOVA), significance <0.05, n = 8/group. Percentage change in different bone and serum parameters in WT and FNDC5 KO female mice with lactation. *=p<0.05 compared to WT. [file elife-92263-supp1.docx]

| Bone Parameters | Virgin | | | | Lactation | | | |
| --- | --- | --- | --- | --- | --- | --- | --- | --- |
|  | WT | | KO | | WT | | KO | |
| **Femoral cortical bone parameters** | | | | | | | | |
| Ct. B. Ar/T. Ar (%) | 47.4 ± 1.2 | | 48 ± 1 | | 35.2 ± 1.8**^a^** | | 37.5 ± 1.8**^b^**^,^ **^c^** | |
| Ct. Th (mm) | 0.18 ± 0.004 | | 0.19 ± 0.005 | | 0.13 ± 0.004**^a^** | | 0.14 ± 0.01**^b^**^,^ **^c^** | |
| Ps. Pm (mm) | 5.16 ± 0.2 | | 5.2 ± 0.06 | | 5.18 ± 0.16 | | 5.2 ± 0.14 | |
| Es. Pm (mm) | 3.95 ± 0.1 | | 4 ± 0.13 | | 4.4 ± 0.11^a^ | | 4.3 ± 0.09^b^ | |
| Marrow cavity area (mm^2^) | 0.93 ± 0.1 | | 0.93 ± 0.04 | | 1.16 ± 0.05^a^ | | 1.13 ± 0.05^b^ | |
| **Femoral trabecular bone parameters** | | | | | | | | |
| BV/TV (%) | 3.7 ± 1 | | 4.5 ± 0.8 | | 3.1 ± 0.7 | | 4 ± 1.1 | |
| Tb. Th (mm) | 0.043 ± 0.002 | | 0.044 ± 0.001 | | 0.039 ± 0.002^a^ | | 0.039 ± 0.001^b^ | |
| Tb. Sp (mm) | 0.37 ± 0.05 | | 0.36 ± 0.03 | | 0.57 ± 0.15^a^ | | 0.44 ± 0.09 | |
| Tb. N (1/mm) | 0.85 ± 0.2 | | 1.06 ± 0.2 | | 0.8 ±0.2 | | 1.04 ± 0.25 | |
| Bone parameters | | Change | | % Change | | | |  |
|  |  |  |  | WT | | KO | |  |
| Cortical Bone Area Fraction | | Decrease | | 26% | | 22% * | |  |
| Cortical Thickness | | Decrease | | 29% | | 24% * | |  |
| Ultimate Force | | Decrease | | 38% | | 31% * | |  |
| Osteoclast Number/ bone parameter | | Increase | | 141% | | 129% | |  |
| TRAP-positive osteocytes | | Increase | | 101% | | 175% * | |  |
| Lacunar Area | | Increase | | 26% | | 15% * | |  |
| Serum RANKL | | Increase | | 170% | | 80% * | |  |

**Supplementary Table 1: FNDC5 KO mice femurs are partially resistant to lactation-induced bone loss.**

Femoral cortical and trabecular bone parameters of WT and FNDC5 KO female virgin and lactation mice. n = 5-8/group. Data presented as mean ± standard deviation. a= significant compared to WT control, b= significant compared to KO control, c= significant compared to WT low Ca diet, 2-way ANOVA, significance <0.05, n= 8/group.

Percentage change in different bone and serum parameters in WT and FNDC5 KO female mice with lactation. *= p<0.05 compared to WT.
