# Supplementary material for: Deletion of FNDC5/irisin modifies murine osteocyte function in a sex-specific manner
Source: eLife. 2024 Apr 25;12:RP92263. doi: 10.7554/eLife.92263 (PMC11045224; doi:10.7554/eLife.92263)
Supplement: Supplementary file 2. — Femoral bone mineral density (BMD), bone mineral content (BMC), cortical and trabecular bone parameters, and mechanical properties of 4- to 5-month-old WT and KO female and male mice under a normal diet or a 2-week low-calcium diet. n = 5/group. Data presented as mean ± standard deviation. a=significant compared to WT control, b=significant compared to KO control, c=significant compared to WT low-calcium diet, two-way analysis of variance (ANOVA), significance <0.05, n = 4–5/group. [file elife-92263-supp2.docx]

| Bone Parameters | Female Normal Diet | | Female Low Ca Diet | | Male Normal Diet | | Male Low Ca Diet | |
| --- | --- | --- | --- | --- | --- | --- | --- | --- |
|  | WT | KO | WT | KO | WT | KO | WT | KO |
| ***Ex vivo* femur DXA** | | | | | | | | |
| BMD (mg/cm^2^) | 75.4± 2.4 | 6.6± 1.5 | 65.4±  4.3**^a^** | 71.4±  3.4 **^c^** | 74.6±  1.5 | 78.3±  3**^a^** | 68.2±  3 | 68.1±  2**^b^** |
| BMC (g) | 0.03± 0.002 | 0.03± 0.001 | 0.024± 0.002**^a^** | 0.027± 0.002 **^b^**^,^**^c^** | 0.029± 0.002 | 0.032± 0.004 | 0.026± 0.002 | 0.025± 0.003**^b^** |
| **Femoral cortical bone parameters** | | | | | | | | |
| Ct.  B.Ar/T.Ar% | 47.8± 1.6 | 48.4± 0.4 | 41.6±  1.1**^a^** | 45.2±  1.4**^b^**^,^ **^c^** | 40.1±  1.4 | 43.6±  0.6**^a^** | 38.3±  0.9 | 39.1±  1.2**^b^** |
| Ct. Th (mm) | 0.2± 0.01 | 0.2± 0.01 | 0.15± 0.01**^a^** | 0.17±  0.01**^b^**^,^ **^c^** | 0.15± 0.01 | 0.2±  0.01**^a^** | 0.14± 0.01 | 0.14± 0.01**^b^** |
| Marrow Cavity Area | 0.92 ± 0.04 | 0.86± 0.02 | 1.02 ± 0.06^a^ | 0.9 ± 0.02^c^ | 1.1 ± 0.04 | 1.03 ± 0.06^a^ | 1.2 ± 0.03 | 1.08 ± 0.03^c^ |
| **Femoral trabecular bone parameters** | | | | | | | | |
| BV/TV (%) | 3.6 ± 1.2 | 4.3 ± 1 | 3.2 ± 1 | 3.9 ± 1 | 6.1 ±  1.1 | 8.7 ± 1.9 | 5.3 ± 1.2 | 6.4 ±0.6 |
| Tb. Th (mm) | 0.059± 0.002 | 0.059± 0.004 | 0.056± 0.002 | 0.055±  0.001 | 0.036 ± 0.001 | 0.035 ± 0.001 | 0.035±0.001 | 0.035 ± 0.002 |
| Tb. Sp (mm) | 0.38 ± 0.03 | 0.35 ± 0.02 | 0.51 ± 0.12^a^ | 0.48 ± 0.08^b^ | 0.274 ± 0.025 | 0.235 ± 0.021 | 0.278 ± 0.027 | 0.265 ± 0.01 |
| Tb. N (1/mm) | 0.81 ± 0.2 | 0.95 ± 0.14 | 0.7 ± 0.02 | 0.91 ±0.13 | 1.7 ± 0.34 | 2.5^a^ ± 0.5 | 1.5 ± 0.3 | 1.8 ± 0.1 |
| **Femoral mechanical properties** | | | | | | | | |
| Ultimate Force (N) | 19±  1 | 19.4± 1.15 | 14.8±  0.7**^a^** | 16.4±  0.5**^b^** | 18.3±  1 | 17.6±  0.9 | 15±  1.3**^a^** | 12.7±  1.5**^b^**^,^ **^c^** |
| Stiffness (N/mm) | 78.6± 3.2 | 79.1± 4.9 | 56.8±  5**^a^** | 67±  4.3 | 76.7±  5.6 | 56.4± 4.75**^a^** | 56±  10.2**^a^** | 48.5±  4.9**^b^**^,^ **^c^** |
| Energy to Failure (N) | 2.9± 0.3 | 3.1±  0.6 | 1.8±  0.5**^a^** | 2±  0.3**^b^** | 3.6±  0.9 | 3.01±  0.6 | 2.5±  0.3**^a^** | 2.35±  0.14 |

**Supplementary Table 2: WT and FNDC5 KO female and male mice bone responds differently to a low-calcium diet**

Femoral BMD, BMC, cortical and trabecular bone parameters, and mechanical properties of 4-5-month-old WT and KO female and male mice under a normal diet or a 2-week low calcium diet. n = 5/group. Data presented as mean ± standard deviation.

a= significant compared to WT control, b= significant compared to KO control, c= significant compared to WT low Ca diet, 2-way ANOVA, significance <0.05, n= 4-5/group.
