# Supplementary material for: Deletion of FNDC5/irisin modifies murine osteocyte function in a sex-specific manner
Source: eLife. 2024 Apr 25;12:RP92263. doi: 10.7554/eLife.92263 (PMC11045224; doi:10.7554/eLife.92263)
Supplement: MDAR checklist [file elife-92263-mdarchecklist1.docx]

**Materials Design Analysis Reporting (MDAR)**

**Checklist for Authors**

**Materials:**

| **Newly created materials** | **Indicate where provided: section/figure legend** | **N/A** |
| --- | --- | --- |
| The manuscript includes a dedicated "materials availability statement" providing transparent disclosure about availability of newly created materials including details on how materials can be accessed and describing any restrictions on access. |  | N/A |
|  |  |  |
| **Antibodies** | **Indicate where provided: section/figure legend** | **N/A** |
| For commercial reagents, provide supplier name, catalogue number and [RRID](https://scicrunch.org/resources), if available. |  | N/A |
|  |  |  |
| **DNA and RNA sequences** | **Indicate where provided: section/figure legend** | **N/A** |
| Genotyping:  WT Forward: GCG GCT CGA GAG ATG AAG AA,  WT Reverse: CAG CCC ACA ACA AGA AGT GC,  KO Forward: GGA CTT CAA GTC CAA GGT CA,  KO Reverse: CCT AAG CCC ACC CAA ATT AC  qPCR:  RANKL (*Tnfsf11*, Forward primer: CCG AGC TGG TGA AGA AAT TAG, Reverse: CCC AAA GTA CGT CGC ATC TTG),  Cathepsin K (*Ctsk*, Primer Bank ID: Mm.PT.58.9655974, IDT),  TRAP (*Acp5*, Mm.PT.58.5755766, IDT),  Sclerostin (*Sost*, Mm00470479_m1, Applied Biosystems)  β-2-microglobulin (*B2m*, Forward: ACA GTT CCA CCC GCC TCA CAT T, Reverse: TAG AAA GAC CAG TCC TTG CTG AAG) | Fig: 1-7 (Genotyped for mice)  Supplementary fig: 3  Real-time quantitative polymerase chain reaction (qPCR) in Methods section |  |
|  |  |  |
| **Cell materials** | **Indicate where provided: section/figure legend** | **N/A** |
| Cell lines: Provide species information, strain. Provide accession number in repository OR supplier name, catalog number, clone number, OR RRID. |  | N/A |
| Primary cultures: Provide species, strain, sex of origin, genetic modification status. |  | N/A |
|  |  |  |
| **Experimental animals** | **Indicate where provided: section/figure legend** | **N/A** |
| Laboratory animals or Model organisms: Mice, C57BL/6J, Female and male, 4-5-months-old, WT and FNDC5 KO (Heterozygous FNDC5 KO mice provided by Dr. Bruce Spiegelman at Harvard University and bred in our facility to obtain homozygous global FNDC5 KO and WT mice). | Fig: 1-7  Animal experiments in Methods section |  |
| Animal observed in or captured from the field: Provide species, sex, and age where possible. |  | N/A |
|  |  |  |
| **Plants and microbes** | **Indicate where provided: section/figure legend** | **N/A** |
| Plants: provide species and strain, ecotype and cultivar where relevant, unique accession number if available, and source (including location for collected wild specimens). |  | N/A |
| Microbes: provide species and strain, unique accession number if available, and source. |  | N/A |
|  |  |  |
| **Human research participants** | **Indicate where provided: section/figure legend) or state if these demographics were not collected** | **N/A** |
| If collected and within the bounds of privacy constraints report on age, sex, gender and ethnicity for all study participants. |  | **N/A** |

**Design:**

| **Study protocol** | **Indicate where provided: section/figure legend** | **N/A** |
| --- | --- | --- |
| If the study protocol has been pre-registered, provide DOI. For clinical trials, provide the trial registration number OR cite DOI.  Animal protocol 20083 | Fig 1-7, Mice used were under this protocol |  |
|  |  |  |
| **Laboratory protocol** | **Indicate where provided: section/figure legend** | **N/A** |
| Provide DOI OR other citation details if detailed step-by-step protocols are available. |  | N/A |
|  |  |  |
| **Experimental study design (statistics details) *** | | |
| **For in vivo studies: State whether and how the following have been done** | **Indicate where provided: section/figure legend. If it could have been done, but was not, write “not done”** | **N/A** |
| Sample size determination: Power analysis | Fig 1-7 |  |
| Randomisation: Mice were grouped in a way so average total body weights among the different groups were not significantly different. | Fig 1-7 |  |
| Blinding: Yes- for data analysis | Fig: 1-7 |  |
| Inclusion/exclusion criteria: During data quality control, one of the KO female control samples (sample 23) was found to have a similar proportion of reads on chromosome Y as in male mice and a very low expression of the gene Xist, typically highly expressed in females (Supplementary Figure 3A, 3B), therefore this sample was excluded from the analysis. | Fig 4-7 |  |
|  |  |  |
| **Sample definition and in-laboratory replication** | **Indicate where provided: section/figure legend** | **N/A** |
| State number of times the experiment was replicated in the laboratory.: 2-3 | Fig 1-7 |  |
| Define whether data describe technical or biological replicates:  Biological | Fig 1-7 |  |
|  |  |  |
| **Ethics** | **Indicate where provided: section/submission form** | **N/A** |
| Studies involving human participants: State details of authority granting ethics approval (IRB or equivalent committee(s), provide reference number for approval. |  | N/A |
| Studies involving experimental animals: State details of authority granting ethics approval (IRB or equivalent committee(s), provide reference number for approval.: IACUC approved protocol 20083. | Animal experiments in Methods section |  |
| Studies involving specimen and field samples: State if relevant permits obtained, provide details of authority approving study; if none were required, explain why. |  | N/A |
|  |  |  |
| **Dual Use Research of Concern (DURC)** | **Indicate where provided: section/submission form** | **N/A** |
| If study is subject to dual use research of concern regulations, state the authority granting approval and reference number for the regulatory approval. |  | N/A |

**Analysis:**

| **Attrition** | **Indicate where provided: section/figure legend** | **N/A** |
| --- | --- | --- |
| Describe whether exclusion criteria were pre-established. Report if sample or data points were omitted from analysis. If yes, report if this was due to attrition or intentional exclusion and provide justification. |  | N/A |
|  |  |  |
| **Statistics** | **Indicate where provided: section/figure legend** | **N/A** |
| When comparing three or more groups with two variables, a two-way analysis of variance (ANOVA) was used.  To compare between two groups, the unpaired, two-tailed Student’s t-test was used.  Differences were considered significant at * p < 0.05, ** p < 0.01, and *** p < 0.001.  For RNA sequence analysis, unadjusted p < 0.01 was used for DEG analysis, and p<0.05 was used for GO analysis.  Data was presented as individual data points. | Statistical Analysis and Differentially expressed gene analysis in Methods section |  |
|  |  |  |
| **Data availability** | **Indicate where provided: section/submission form** | **N/A** |
| For newly created and reused datasets, the manuscript includes a data availability statement that provides details for access (or notes restrictions on access). | Data Availability Statement Section |  |
| When newly created datasets are publicly available, provide accession number in repository OR DOI and licensing details where available.  Osteocyte transcriptome data has been deposited into the NCBI GEO database. The accession number for the data is GSE242445. | Data Availability Statement Section |  |
| If reused data is publicly available provide accession number in repository OR DOI, OR URL, OR citation. |  | N/A |
|  |  |  |
| **Code availability** | **Indicate where provided: section/figure legend** | **N/A** |
| For any computer code/software/mathematical algorithms essential for replicating the main findings of the study, whether newly generated or re-used, the manuscript includes a data availability statement that provides details for access or notes restrictions. |  | N/A |
| Where newly generated code is publicly available, provide accession number in repository, OR DOI OR URL and licensing details where available. State any restrictions on code availability or accessibility. |  | N/A |
| If reused code is publicly available provide accession number in repository OR DOI OR URL, OR citation. |  | N/A |

**Reporting:**

The MDAR framework recommends adoption of discipline-specific guidelines, established and endorsed through community initiatives.

| **Adherence to community standards** | **Indicate where provided: section/figure legend** | **N/A** |
| --- | --- | --- |
| State if relevant guidelines (e.g., ICMJE, MIBBI, ARRIVE, STRANGE) have been followed, and whether a checklist (e.g., CONSORT, PRISMA, ARRIVE) is provided with the manuscript. |  | N/A |
